# Extended-Infusion β-Lactam Therapy, Mortality, and Subsequent Antibiotic Resistance Among Hospitalized Adults With Gram-Negative Bloodstream Infections

**DOI:** 10.1001/jamanetworkopen.2024.18234

**Published:** 2024-07-02

**Authors:** Sara M. Karaba, Sara E. Cosgrove, Jae Hyoung Lee, Suiyini Fiawoo, Emily L. Heil, Katelyn S. Quartuccio, Katherine C. Shihadeh, Pranita D. Tamma

**Affiliations:** 1 Department of Medicine, Division of Infectious Diseases, Johns Hopkins University School of Medicine, Baltimore, Maryland; 2 Department of Practice, Sciences, and Health-Outcomes Research, University of Maryland School of Pharmacy, Baltimore, Maryland; 3 Department of Pharmacy, University of Rochester Medical Center, Rochester, New York; 4 Department of Pharmacy, Denver Health Medical Center, Denver, Colorado; 5 Department of Pediatrics, Johns Hopkins University School of Medicine, Baltimore, Maryland

## Abstract

**Importance:**

Current evidence is conflicting for associations of extended-infusion β-lactam (EI-BL) therapy with clinical outcomes.

**Objective:**

To investigate the association of EI-BL therapy with survival, adverse events, and emergence of antibiotic resistance in adults with gram-negative bloodstream infections (GN-BSI).

**Design, Setting, and Participants:**

This cohort study of consecutive adults with GN-BSI admitted to 24 United States hospitals between January 1, 2019, and December 31, 2019, receiving EI-BL were compared with adults with GN-BSI receiving the same agents as intermittent infusion β-lactam (II-BL; ≤1-hour infusions). Statistical analysis was performed from January to October 2023.

**Exposures:**

EI-BL (ie, ≥3-hour infusion).

**Main Outcomes and Measures:**

EI-BL and II-BL groups underwent 1:3 nearest-neighbor propensity score matching (PSM) without replacement. Multivariable regression was applied to the PSM cohort to investigate outcomes, all censored at day 90. The primary outcome was mortality; secondary outcomes included antibiotic adverse events and emergence of resistance (≥4-fold increase in the minimum inhibitory concentration of the β-lactam used to treat the index GN-BSI).

**Results:**

Among the 4861 patients included, 2547 (52.4%) were male; and the median (IQR) age was 67 (55-77) years. There were 352 patients in the EI-BL 1:3 PSM group, and 1056 patients in the II-BL 1:3 PSM group. Among 1408 PSM patients, 373 (26.5%) died by day 90. The odds of mortality were lower in the EI-BL group (adjusted odds ratio [aOR], 0.71 [95% CI, 0.52-0.97]). In a stratified analysis, a survival benefit was only identified in patients with severe illness or elevated minimum inhibitory concentrations (ie, in the intermediate range for the antibiotic administered). There were increased odds of catheter complications (aOR, 3.14 [95% CI, 1.66-5.96]) and antibiotic discontinuation because of adverse events (eg, acute kidney injury, cytopenias, seizures) in the EI-BL group (aOR, 3.66 [95% CI, 1.68-7.95]). Emergence of resistance was similar in the EI-BL and II-BL groups at 2.9% vs 7.2%, respectively (*P* = .35).

**Conclusions and Relevance:**

In this cohort study of patients with GN-BSI, EI-BL therapy was associated with reduced mortality for patients with severe illness or those infected with nonsusceptible organisms; potential advantages in other groups remain unclear and need to be balanced with potential adverse events. The subsequent emergence of resistance warrants investigation in a larger cohort.

## Key Points

**Question:**

Is extended-infusion β-lactam (EI-BL) therapy associated with mortality, adverse events, and the subsequent emergence of antibiotic resistance in patients with gram-negative bloodstream infections (GN-BSI)?

**Findings:**

In this cohort study of 4861 adults with GN-BSI, EI-BL therapy was associated with reduced mortality among patients with severe illness and/or antibiotic minimum inhibitory concentrations in the intermediate range to the β-lactam agent administered for treatment. There were increased odds of catheter complications and antibiotic discontinuation due to adverse events in the EI-BL group; and the emergence of antibiotic resistance was similar in the EI-BL and intermittent infusion β-lactam groups.

**Meaning:**

These results suggest that patients with GN-BSI who are severely ill or infected with bacteria with elevated antibiotic minimum inhibitory concentrations may benefit from EI-BL therapy.

## Teaser

This cohort study assesses whether extended-infusion β-lactam therapy is associated with survival, adverse events, and/or emergence of resistance in adults with gram-negative bloodstream infections.

## Introduction

β-lactam agents remain the mainstay of treatment for gram-negative bloodstream infections (GN-BSI). Extending the infusion of β-lactams (ie, continuous infusion or extended-infusion β-lactam [EI-BL] therapy) increases the likelihood of achieving adequate serum drug levels (ie, free time of the β-lactam agent above the organism minimum inhibitory concentration [MIC]).^1,2,3,4^ Pharmacokinetic and pharmacodynamic studies indicate that extending the infusion of a β-lactam agent may be particularly beneficial for gram-negative organisms with elevated antibiotic MICs.^1^ Several observational studies^5,6,7,8,9,10,11^ and clinical trials^12,13,14,15,16,17,18,19,20,21^ have investigated the effect of EI-BL therapy on patient outcomes. Clinical trials, however, have shown conflicting results with some suggesting no benefit^12,13,14,17,18,20,21^ and others indicating improved patient outcomes^11,15,16,19^ with EI-BL therapy. A notable limitation to some of the most well-known trials is that relatively few patients (approximately 20%) had confirmed gram-negative bacterial infections, obscuring accurate estimates of the effect of EI-BL on clinical outcomes of infected patients.^19,20,21^ Due to incongruous results, a clinical trial estimated to enroll approximately 7000 patients randomized to EI-BL and intermittent infusion β-lactams (II-BL) is currently under way.^22^ To our knowledge, no clinical trial has evaluated the effect of EI-BL therapy on the subsequent emergence of antimicrobial resistance.

We previously conducted a single-center study that suggested that extending the infusion of β-lactam administration was associated with reduced risk for the emergence of subsequent resistance.^23^ It is plausible that sustained serum antibiotic concentrations associated with EI-BL may reduce the opportunity for bacteria to acquire resistance. This investigation needs to be repeated in a larger cohort as extending the infusion of β-lactam therapy to prevent subsequent resistance, if effective, could be a relatively non–resource intensive clinical intervention. In the present study, we sought to evaluate the association of EI-BL therapy with clinical outcomes, adverse events, and the emergence of resistance in a multicenter cohort of adult patients with GN-BSI.

## Methods

### Setting and Participants

We conducted a retrospective cohort study of all unique, consecutive patients 18 years of age and older with monomicrobial GN-BSI hospitalized at 1 of 24 United States hospitals in 2019.^24^ The institutional review boards of each participating site approved the study with a waiver of informed consent as treatment decisions were at the discretion of the inpatient treatment team and all data were collected retrospectively. Johns Hopkins University served as the central Data Coordinating and Statistical Analysis Center. A waiver of informed consent was granted by Johns Hopkins University as it was determined that the research posed minimal risk as there was no direct patient contact by members of the study team. The Strengthening the Reporting of Observational Studies in Epidemiology (STROBE) reporting guideline was followed.^25^

### Data Extraction

Infectious disease specialists collected or closely oversaw all data collection. The following data were collected: demographics; preexisting medical conditions; severity of illness; source of BSI and source control measures; bacterial species and MIC data; detailed information about the dosing, infusion time, and duration of antibiotics administered; and clinical outcomes data. Clinical and Laboratory Standards Institute antibiotic MIC interpretive criteria were applied to determine susceptibility to treatment agents.^26^

### Exposed and Unexposed Groups

The exposed group included patients who received EI-BL therapy for at least 72 hours. EI-BL was defined as the administration of a β-lactam agent over 3 or more hours, and included agents frequently administered using an EI strategy (ie, piperacillin-tazobactam, aztreonam, ceftazidime, cefepime, meropenem, imipenem-cilastatin, ceftazidime-avibactam, ceftolozane-tazobactam, meropenem-vaborbactam, imipenem-cilastatin-relebactam, or cefiderocol). Unexposed patients were those who received intravenous β-lactam therapy over 1 hour or less (ie, II-BL). All antibiotic therapy plans were at the discretion of the clinical team.

### Outcomes

The primary outcome was mortality within 90 days of the day of blood culture collection. Secondary outcomes included the following: (1) recurrent infection with the same bacterial species, (2) the emergence of an antibiotic-resistant organism of the same bacterial species as the index GN-BSI, and (3) treatment-associated adverse events, all censored at 90 days. The 90-day outcomes were selected to align with the ongoing clinical trial investigating EI-BL vs II-BL therapy.^22^

A recurrent infection was defined as growth of the same bacterial species as the index GN-BSI, from any source, with at least a 7-day blood culture negative interval from the last positive culture. Emergence of antibiotic resistance was defined as a 4-fold or greater increase in the MIC of the β-lactam agent used to treat the index GN-BSI.^23^ For example, if the cefepime MIC against *Escherichia coli* identified in a blood culture on day 1 was 2 μg/mL and the patient was treated with cefepime, *E coli* recovered on or after day 8 in a clinical culture with a cefepime MIC of 8 μg/mL or higher would qualify as meeting the end point of emergence of resistance.

Mortality and recurrent infection were further investigated in 2 stratified subgroup analyses established a priori: (1) patients with vs without a Pitt bacteremia score of at least 4 points^27^ and (2) patients infected with organisms with β-lactam MICs in the intermediate or susceptible dose-dependent range who received the same β-lactam as therapy (herein, referred to as the elevated MIC group) vs patients infected with gram-negative isolates with a β-lactam MIC in the susceptible range who received the same β-lactam as therapy.

Treatment-associated adverse events included any vascular catheter (eg, midline, peripherally inserted central catheter, central line, implanted port) complications (eg, line malfunction necessitating an intervention, phlebitis, secondary catheter-related bloodstream infection) while receiving antibiotic therapy for the GN-BSI or any incident adverse events (eg, acute kidney injury, allergic reaction, rash, hepatotoxicity^28^) that led to early discontinuation of the β-lactam agent administered for the index infection.

### Definitions

The Pitt bacteremia score consisted of the following components: temperature derangements, hypotension, mechanical ventilation, cardiac arrest, and mental status changes.^27^ Severe immunocompromise was defined by at least 1 of the following: hematopoietic stem cell transplant in the prior 12 months or active treatment for graft-vs-host disease, solid organ transplant recipient, malignant neoplasm with active chemotherapy in the prior 3 months, neutropenia (absolute neutrophil count <500 cells/mm^3^), HIV with CD4 count less than 200 cells/mm^3^, or receipt of corticosteroids at a dose equivalent to 10 mg daily of prednisone for more than 14 days or receipt of biologic medications. Active antibiotic therapy was defined as receipt of an antibiotic agent on day 1 of GN-BSI to which the bacterial isolate was susceptible, according to CLSI criteria.^26^

### Statistical Analysis

Baseline data were compared using the Pearson χ^2^ test for categorical variables or the Wilcoxon rank-sum test for continuous variables. To ensure patients in the exposed group (ie, EI-BL therapy) and unexposed group (ie, II-BL therapy) were as similar as possible, propensity score matching (PSM) was used. Propensity scores were developed using multivariable logistic regression to model the odds of receiving EI-BL vs II-BL therapy for GN-BSI. Covariates included in generating propensity scores were selected a priori based on hypothesized associations and included the following: (1) Pitt bacteremia score greater than or equal to 4, (2) Charlson Comorbidity Index greater than or equal to 5, (3) intensive care unit admission on day 1, (4) severe immune compromise, (5) adequate source control by day 14, (5) receipt of active antibiotic therapy on day 1, (6) urinary source of BSI, and (7) the 3 most common bacterial species identified (ie, *E coli* BSI, *Klebsiella pneumoniae* BSI, and *Pseudomonas aeruginosa* BSI).

We used 1:3 nearest neighbor PSM without replacement. Adequacy of matching between exposed and unexposed groups was assessed using standardized mean difference (SMD), with SMDs less than 0.20 representative of an adequate match. Outcomes in the 1:3 PSM population were evaluated using multivariable logistic regression. Patients eligible for inclusion in each of the stratified analyses underwent 1:3 PSM, incorporating the aforementioned variables, except for the variable informing stratification (eg, Pitt bacteremia score was not included as a matching variable in the subgroup analysis comparing the outcomes of patients receiving EI-BL vs II-BL with Pitt bacteremia scores ≥4). Covariates used in generating propensity scores were included in models to increase robustness.^29^ Odds ratios (ORs), 95% CIs, and *P* values were estimated using cluster robust standard errors. Two-sided *P *<* *.05 was considered statistically significant for all tests. Statistical analysis was completed using Stata version 16.1 (StataCorp) and R version 4.2.2 (R Project for Statistical Computing), using the MatchIt package. Data analysis was performed from January to October 2023.

## Results

Among the 4861 unique patients with GN-BSI included in the cohort, 2547 (52.4%) were male; 352 (7.2%) received EI-BL therapy and 4509 (92.7%) received II-BL therapy; and the median (IQR) age was 67 (55-77) years. Baseline characteristics in the full, unmatched cohort are listed in Table 1.

**Table 1.** Baseline Characteristics of Patients Receiving Extended-Infusion vs Intermittent Infusion β-Lactam Therapy for Gram-Negative Bloodstream Infections, Before and After Propensity Score Matching

Characteristic	Total (N = 4861)	Full cohort			Propensity-score matched cohort		
		Extended infusion (n = 352)	Intermittent infusion (n = 4509)	*P* value	Extended infusion (n = 352)	Intermittent infusion (n = 1056)	Standardized mean difference
Age, median (IQR), y	67 (55-77)	63 (52-71.5)	67 (55-77)	<.001	NA	NA	NA
Sex, No. (%)^a^							
Male	2547 (52.4)	208 (59.1)	2339 (51.9)	.01	NA	NA	NA
Female	2303 (47.3)	144 (40.9)	2159 (47.9)	.01			
Pitt bacteremia score ≥4, No. (%)	946 (19.5)	93 (26.4)	853 (18.9)	.001	93 (26.4)	292 (27.7)	−0.0279
Charlson comorbidity index ≥5, No. (%)	738 (15.2)	48 (13.6)	690 (15.3)	.40	48 (13.6)	141 (13.4)	0.0083
Severe immune compromise, No. (%)^b^	1419 (29.2)	134 (38.1)	1285 (28.5)	<.001	134 (38.1)	386 (36.6)	0.0312
Solid organ transplant, No. (%)	299 (6.2)	36 (10.2)	263 (5.8)	.001	NA	NA	NA
Bone marrow transplant, No. (%)	106 (2.2)	10 (2.8)	96 (2.1)	.38	NA	NA	NA
ANC<500 cells/μL, No. (%)	299 (6.2)	27 (7.7)	272 (6.0)	.22	NA	NA	NA
ICU, No. (%)	1574 (32.4)	163 (46.3)	1411 (31.3)	<.001	163 (46.3)	491 (46.5)	−0.0038
Source controlled by day 14, No. (%)^c^	4077 (83.9)	263 (74.7)	3814 (84.6)	<.001	263 (74.7)	785 (74.3)	0.0087
Source of BSI, No. (%)							
Intra-abdominal^d^	1250 (25.7)	127 (36.1)	1123 (24.9)	<.001	NA	NA	NA
Neutropenic fever^e^	119 (2.4)	10 (2.8)	109 (2.4)	.62	NA	NA	NA
Prostatitis	45 (0.9)	1 (0.3)	44 (1.0)	.19	NA	NA	NA
Respiratory	272 (5.6)	29 (8.2)	243 (5.4)	.03	NA	NA	NA
Skin or soft tissue	227 (4.7)	23 (6.5)	204 (4.5)	.09	NA	NA	NA
Urinary tract	2321 (47.7)	97 (27.6)	2224 (49.3)	<.001	97 (27.6)	286 (27.1)	0.0106
Vascular catheter	323 (6.6)	41 (11.6)	282 (6.3)	<.001	NA	NA	NA
Active empirical antibiotic therapy day 1, No. (%)	4320 (88.9)	318 (90.3)	4002 (88.8)	.36	318 (90.3)	972 (92.0)	−0.0557
Microbiology of BSI							
* Escherichia coli*	2471 (50.8)	139 (39.5)	2332 (51.7)	<.001	139 (39.5)	420 (39.8)	−0.0058
* Klebsiella pneumoniae*	841 (17.3)	59 (16.8)	782 (17.3)	.78	59 (16.8)	190 (18.0)	−0.0330
* Pseudomonas aeruginosa*	423 (8.7)	48 (13.6)	375 (8.3)	.001	48 (13.6)	125 (11.8)	0.0524

Abbreviations: ANC, absolute neutrophil count; BSI, bloodstream infection; ICU, intensive care unit; NA, not applicable.

^a^ Eleven patients were missing data for the sex variable.

^b^ Severe immune compromise defined by at least 1 of the following: hematopoietic stem cell transplant in the prior 12 months or active treatment for graft-vs-host disease, solid organ transplant recipient, malignant neoplasm with active chemotherapy in the prior 3 months, neutropenia (absolute neutrophil count <500 cells/mm^3^), HIV with CD4 count greater than 200 cells/mm^3^, or receipt of corticosteroids at a dose equivalent to 10 mg daily of prednisone for more than 14 days or other immunosuppressive therapy.

^c^ Source control was needed and achieved (eg, removal of infected catheter, drainage of fluid collection).

^d^ Intra-abdominal includes intra-abdominal abscess, typhlitis, presumed translocation in patients with diarrhea or other intestinal issues, and hepatobiliary including hepatic abscess.

^e^ Neutropenic fever with no other identified source.

The most common bacterial species were *E coli* (50.8% [2471 of 4861]), *K pneumoniae* (17.3% [841 of 4861]), and *P aeruginosa* (8.7% [423 of 4861]). The median (IQR) duration of bacteremia was 1 (1-1) day in both groups. Patients in the EI-BL group were more likely to have severe immunocompromise (38% vs 29%; *P* < .001), to be in the ICU (46% vs 31%; *P* < .001), and to have a Pitt bacteremia score of at least 4 (26% vs 19%; *P* = .001). Patients in the EI-BL group were less likely to have a urinary source of their GN-BSI (28% vs 49%; *P* < .001) and were less likely to have adequate source control (75% vs 85%; *P* < .001). Overall, these findings suggest that in the full cohort, patients receiving EI-BI were generally more critically ill and had more complex comorbidities compared with patients receiving II-BL therapy.

The 1:3 PSM cohort consisted of 352 patients (25%) receiving EI-BL and 1056 (75%) receiving II-BL. Baseline differences observed in the full cohort were no longer present in the PSM cohort; the SMD for all variables was less than 0.20 (Table 1 and Figure). The median (IQR) duration of EI-BL therapy was 4 (3-9) days. The median (IQR) duration of antibiotic therapy in the PSM cohort was 11 (9-14) days, similar between both treatment groups.

**Figure.**
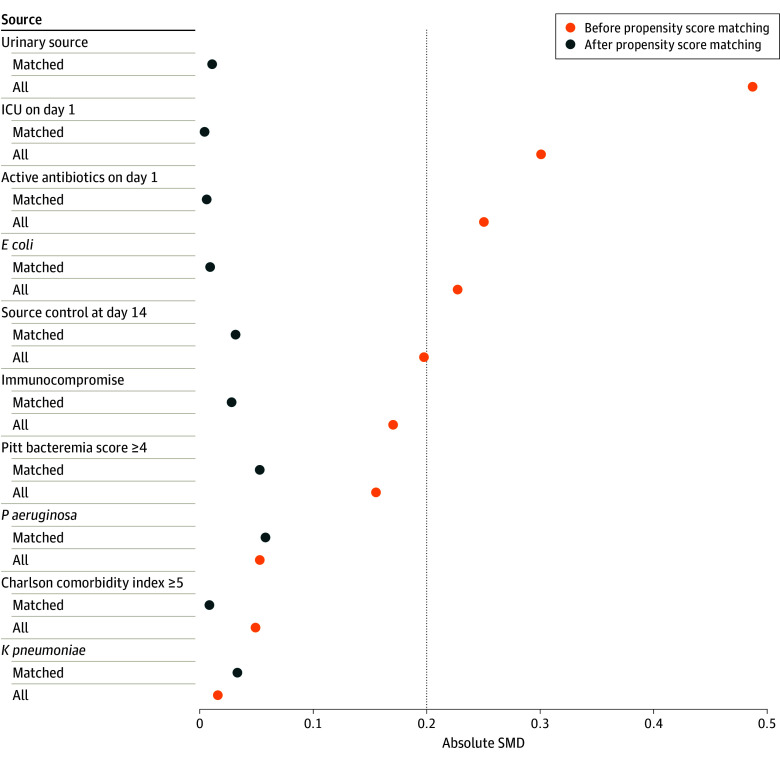
Standardized Mean Differences (SMDs) Before and After Propensity Score Matching Using 3:1 Nearest Neighbor Without Replacement *E coli* indicates *Escherichia coli*; ICU, intensive care unit; *K pneumoniae*, *Klebsiella pneumoniae*; *P aeruginosa*, *Pseudomonas aeruginosa*.

### Ninety-Day Mortality

There were 79 deaths (22%) in the EI-BL group, and 294 deaths (28%) in the II-BL group (adjusted odds ratio [aOR], 0.71, 95% CI [0.52-0.97]; *P* = .03) (Table 2). Mortality was further investigated in 2 stratified subgroup analyses: (1) patients with and without a Pitt bacteremia score of at least 4 points^27^ and (2) the elevated MIC group. Baseline variables were well-balanced in both subgroups that underwent stratified analyses (eAppendix in Supplement 1).

**Table 2.** Ninety-Day Clinical Outcomes in a Propensity Score-Matched Cohort of Adults With Gram-Negative Bloodstream Infections^a^

Outcome	Extended infusion (n = 352 [25%])	Intermittent infusion (n = 1056 [75%])	aOR (95% CI)	*P* value^b^
Mortality, No. (%)	79 (22.4)	294 (27.8)	0.71 (0.52-0.97)	.03
Recurrent infection, No. (%)	35 (9.9)	111 (10.5)	0.96 (0.64-1.45)	.85
Emergence of a subsequent antibiotic resistant infection, No. (%)^c^	1 (2.9)^d^	8 (7.2)^d^	NA	NA
Vascular catheter complication, No. (%)	21 (6.0)	21 (2.0)	3.14 (1.66-5.96)	<.001
Early antibiotic discontinuation because of an antibiotic-associated adverse event, No. (%)	15 (4.3)	13 (1.2)	3.66 (1.68-7.95)	.001
Subgroup of patients with Pitt bacteremia score ≥4 (n = 372)				
Mortality, No. (%)	36 (38.7)	155 (55.6)	0.47 (0.28-0.81)	.006
Recurrent infection, No. (%)	11 (11.8)	33 (11.8)	1.05 (0.49-2.22)	.91
Subgroup of patients with Pitt bacteremia score <4 (n = 956)				
Mortality, No. (%)	42 (17.6)	129 (17.9)	0.93 (0.62-1.38)	.71
Recurrent infection, No. (%)	21 (8.8)	78 (10.9)	0.81 (0.47-1.37)	.42
Subgroup of patients with bacterial isolates with elevated β-lactam MICs and receipt of that β-lactam agent (n = 112)^e^				
Mortality, No. (%)	2 (7.1)	20 (23.8)	0.06 (0.01-0.66)	.02
Recurrent infection, No. (%)	3 (10.7)	7 (8.3)	2.96 (0.30-29.9)	.36
Subgroup of patients with bacterial isolates with susceptible β-lactam MICs^e^ and receipt of that β-lactam agent (n = 1296)				
Mortality, No. (%)	77 (23.8)	274 (28.1)	0.75 (0.54-1.05)	.10
Recurrent infection, No. (%)	32 (9.9)	112 (11.5)	0.86 (0.56-1.32)	.49

Abbreviations: aOR, adjusted odds ratio; MIC, minimum inhibitory concentration; NA, not applicable.

^a^ Comparing receipt of extended-infusion β-lactam to intermittent infusion; additional adjustment for covariates used to generate propensity scores.

^b^ *P* value for the adjusted odds ratio.

^c^ Defined as a subsequent infection with the same bacterial species with at least a 4-fold increase in the β-lactam MIC of the β-lactam agent administered to treat the index gram-negative bloodstream infection.

^d^ Extended infusion (n = 35); intermittent infusion (n = 111).

^e^ Defined as antibiotic MICs in the intermediate or susceptible dose-dependent range for the antibiotic administered.

Patients with a Pitt bacteremia score of at least 4 points had significantly lower odds of mortality if receiving EI-BL compared with II-BL therapy (aOR, 0.47 [95% CI, 0.28-0.81]; *P* = .006). For patients with a Pitt bacteremia score of less than 4 points, a survival benefit was not observed in the EI-BL group (aOR, 0.93 [95% CI, 0.62-1.38]; *P* = .71).

Among patients in the elevated β-lactam MIC group, treatment with EI-BL compared with II-BL led to a decrease in the odds of mortality (aOR, 0.06 [95% CI, 0.01-0.66]; *P* = .02). Mortality was similar regardless of receipt of EI-BL or II-BL therapy among patients infected with organisms with antibiotic MICs in the susceptible range (aOR, 0.75 [95% CI, 0.54-1.05]; *P* = .10).

### Recurrent Infection

In the PSM cohort, 35 of 352 patients (9.9%) in the EI-BL group and 111 of 1056 (10.5%) in the II-BL group experienced a recurrent infection (Table 2). There was no difference in the odds of recurrent infection between the groups (aOR, 0.96 [95% CI, 0.64-1.45]; *P* = .86). Differences in the odds of recurrent infection were also not significant when the analysis was limited to patients with a Pitt bacteremia score of at least 4 points or those infected with organisms with elevated β-lactam MICs.

### Emergence of Antibiotic Resistance

An analysis of the emergence of antibiotic resistance comparing the EI-BL and II-BL groups was limited to patients who had a recurrent infection with the same bacterial species within 90 days. In the PSM population, 1 of 35 (2.9%) patients in the EI-BL group experienced a subsequent infection for which the β-lactam MIC for the antibiotic administered for the index infection increased by at least 4-fold. In contrast, 8 of 111 patients (7.2%) in the II-BL group had a subsequent infection with at least a 4-fold increase in the MIC, but this was not a statistically significant difference (*P* = .35).

### Treatment-Associated Adverse Events

In the PSM cohort, 65 of 1408 patients (5%) experienced a treatment-associated adverse event, including 33 of 352 (9%) in the EI-BL group and 32 of 1056 (3%) in the II-BL group. More specifically, 21 (6%) and 21 (2%) patients experienced a vascular catheter complication in the EI-BL and II-BL groups, respectively (aOR, 3.14 [95% CI, 1.66-5.96]; *P* < .001). Fifteen of 352 patients (4%) in the EI-BL group and 13 of 1056 patients (1%) in the II-BL group had early cessation of a β-lactam antibiotic because of a likely antibiotic-associated adverse event (aOR, 3.66 [95% CI, 1.68-7.95]; *P* = .001).

## Discussion

We identified a survival benefit among adult patients with GN-BSI receiving EI-BL compared with II-BL therapy. The benefit was largely associated with critical illness (ie, Pitt bacteremia score ≥4) and GN-BSI with elevated antibiotic MICs (ie, intermediate or susceptible dose-dependent range). In a stratified analysis excluding these subgroups, EI-BL was no longer associated with a survival benefit. The potential benefits of EI-BL therapy need to be balanced against the increased risk of complications that may be associated with this infusion strategy. We found an increased risk of both vascular catheter-related complications and antibiotic treatment-related complications in the EI-BL group compared with the II-BL group. Taken together, these results suggest that while EI-BL therapy may be associated with positive outcomes, the benefits may not surpass the risks if applied to all patients. Rather, a more targeted approach may be necessary for patients who are severely ill or those known to have or be at reasonable risk for infection with an elevated β-lactam MIC.

Although some observational studies have demonstrated a survival benefit with EI-BL therapy compared with II-BL therapy,^30,31^ the same has not been observed in clinical trials.^19,20,21^ Between 2013 and 2016, 3 key randomized clinical trials investigated the outcomes of critically ill patients receiving EI-BL (ie, continuous infusion β-lactam therapy) or II-BL therapy.^19,20,21^ Individually, these studies were not powered to investigate mortality as a primary outcome and none of these trials demonstrated reduced mortality with EI-BL therapy. An important consideration in reviewing the 3 trials^19,20,21^ is that only approximately 20% of patients had a gram-negative organism recovered. In the absence of a confirmed bacterial infection, antibiotics, regardless of whether administered intermittently or via prolonged infusion, are unlikely to improve patient outcomes. Moreover, even for confirmed infections, antibiotic MIC data were largely unavailable,^19,20,21^ precluding subgroup analyses of patients infected with organisms with elevated MICs.

A meta-analysis evaluated the combined findings of the 632 patients across the 3 aforementioned trials and identified in-hospital mortality to be significantly lower in the EI-BL group compared with the II-BL group, at 20% and 26%, respectively.^32^ Other meta-analyses have identified similar survival benefits with EI-BL use.^33,34^ A subsequent clinical trial not included in these meta-analyses enrolled 607 critically ill adults between 2018 and 2022 across 31 ICUs receiving EI-BL (ie, continuous-infusion meropenem) compared with II-BL therapy (ie, II-meropenem) and found that 90-day mortality was identical between the treatment groups at 42%.^35^ Although a higher portion of patients (77%) had a confirmed gram-negative pathogen compared with prior trials, the majority (87%) were from nonsterile sites and 32% were resistant to carbapenems, favoring the likelihood of the null hypothesis being accepted. Finally, in a propensity-score analysis of 224 patients with GN-BSI (ie, all patients had confirmed invasive gram-negative infections), there was a 54% reduction in the risk of mortality in patients receiving EI-BL therapy, compared with II-BL therapy.^36^

In our study, a survival benefit was limited to critically ill patients with documented GN-BSI and to patients with GN-BSI with elevated β-lactam MICs. We believe that a benefit in both of these subgroups is biologically plausible. Regarding the former, critically ill patients display alterations in pharmacokinetics that lead to unpredictable and often subtherapeutic antibiotic concentrations. This pharmacokinetic variability has been shown to be reduced with prolonged infusion strategies.^21,37,38^ For patients with higher MIC infections, dose-optimization schemes such as prolonged-infusion strategies are generally necessary to reliably achieve standard pharmacodynamic targets.^39^

Unfortunately, our study was not powered to investigate the effect of EI-BL therapy on the subsequent emergence of resistance as only approximately 10% of patients had a recurrent infection with the same bacterial species. Available studies that have attempted to address this question are somewhat limited as few patients had documented bacterial infections and even fewer had recurrent infections.^19,20,21,35^ Moreover, inconsistent definitions of resistance were used.^11,35,40,41^ In some cases, development of resistance was defined as resistance to specific classes of antibiotics, whereas in other studies it was defined as a change from susceptible to nonsusceptible for a particular drug (and not necessarily to the antibiotic administered for the initial infection). Furthermore, it is often not clear whether subsequent cultures were obtained based on clinical need or surveillance purposes, with the latter likely being less consequential for patients.^35,40,41^ In contrast, we elected to focus on the emergence of resistance to the antibiotic prescribed for the index infection. We realize our definition was very restrictive on the number of evaluable patients; however, we believe a rigorous definition of resistance is needed to appropriately investigate this question.

A notable finding in this study was the increased risk of treatment-related adverse events (both catheter-associated and antibiotic-associated) in the EI-BL group. Patients receiving EI-BL may require a dedicated vascular catheter and possibly more than 1 catheter, increasing the likelihood of catheter-associated complications. Moreover, as EI-BL strategies often use higher daily drug dosages (eg, 6 vs 3 total daily grams of meropenem), this approach may increase the likelihood of antibiotic-associated adverse events such as cytopenias, seizures, and acute kidney injury.

### Limitations

This study has limitations. First, 24 sites collected data, thus there may be inaccuracies in manual data entry. Mitigation strategies included data collection performed primarily by trained infectious disease specialists or through closely supervised trainees. Moreover, a detailed data dictionary was developed, regular virtual meetings were held across sites to ensure consistent data collection practices, and thorough data cleaning with queries to sites as needed for outlying or missing data was conducted prior to analysis. Second, these data reflect GN-BSIs in 2019 and the use of EI-BL therapy in 2019 may not be reflective of current practices. In a survey conducted in 2021, critical care and infectious disease specialists from 409 hospitals acknowledged the use of EI-BL infusion strategies for approximately 50% of ICU patients, somewhat comparable with the 46% in our cohort.^42^ Similar data outside of ICU settings are not available. Third, this is an observational study, thus there are likely confounding variables which may be unaccounted for and which may have influenced the decision of which infusion strategy to use. In an attempt to limit confounding by indication (eg, a patient with more severe illness and medical complexity may be more likely to receive EI-BL therapy), PSM was used to develop 2 well-matched groups, where the only clear difference was the infusion strategy. While this strategy precluded using data from all 4861 patients in the overall cohort, it increased the likelihood that the 2 groups being compared were similar based upon factors which could affect outcomes such as severity of illness, comorbidities, source of infection, source control, active antibiotic therapy, and causative organisms. While PSM enables comparison between 2 similar groups, it does not eliminate selection bias. Fourth, attributing antibiotic adverse events to a specific antibiotic can be challenging. For example, if someone received EI-BL therapy with cefepime on days 1 to 5, transitioned to oral ciprofloxacin and on day 8, and developed a *Clostridioides difficile* infection on day 11, in our study this would be attributed to the EI-BL group, even though it may have been due to or exacerbated with ciprofloxacin. Additionally, patients who received EI-BL therapy generally did not receive this antibiotic administration strategy for the full treatment course. However, as EI-BL administration is often impractical after hospital discharge, this is reflective of clinical practice.

## Conclusions

This cohort study’s results suggest that the populations that benefit most from EI-BL therapy are those who are critically ill or infected with organisms with elevated MICs. To balance the potential adverse events associated with EI-BL (ie, both from vascular catheters and from antibiotics), the potential advantages of EI-BL therapy should be weighed against potential harm in all other populations. Whether or not EI-BL may reduce the development of future antibiotic resistance remains unanswered and warrants further investigation.

## Notes

Supplement 1. **eAppendix.** Supplemental Material

Supplement 2.
Data Sharing Statement
