# Supplementary material for: Extended-Infusion β-Lactam Therapy, Mortality, and Subsequent Antibiotic Resistance Among Hospitalized Adults With Gram-Negative Bloodstream Infections
Source: JAMA Netw Open. 2024 Jul 2;7(7):e2418234. doi: 10.1001/jamanetworkopen.2024.18234 (PMC11220563; doi:10.1001/jamanetworkopen.2024.18234)
Supplement: Supplement 1. — eAppendix. Supplemental Material [file jamanetwopen-e2418234-s001.pdf]

Supplemental Online Content

Karaba SM, Cosgrove SE, Lee JH, et al. Extended-infusion β-lactam therapy, mortality, and subsequent antibiotic resistance among hospitalized adults with gram-negative bloodstream infections. *JAMA Netw Open.* 2024;7(7):e2418234. doi:10.1001/jamanetworkopen.2024.18234

eAppendix. Supplemental Material

This supplemental material has been provided by the authors to give readers additional information about their work.

© 2024 Karaba SM et al. *JAMA Network Open*.

eAppendix. Supplemental Material

Supplemental Table 1. Baseline characteristics of subgroup of patients with Pitt bacteremia score ≥4 patients receiving extended-infusion versus intermittent infusion beta-lactam therapy for gram-negative bloodstream infections, before and after propensity-score matching

		**Full Cohort**			**Propensity-Score Matched Cohort**		
	Total n= 946	Extended infusion n=93	Intermittent Infusion n=853	P-value	Extended infusion n=93	Intermittent Infusion n=279	Standardized Mean Difference
Age, median (IQR)	67 (56-77)	60 (50-73)	68 (57-77)	0.004	NA[f]	NA	NA
Sex[a]					NA	NA	NA
Male, n (%)	512 (54.1)	57 (61.3)	455 (53.3)	0.31	NA	NA	NA
Female, n (%)	431 (45.6)	36 (38.7)	395 (46.3)	0.31			
Pitt bacteremia score ≥4, n (%)	946	NA	NA	NA	NA	NA	NA
Charlson comorbidity index ≥5, n (%)	151 (16.0)	14 (15.1)	137 (16.1)	0.12	14 (15.1)	39 (14.0)	0.0301
Severe immune compromise, n (%)[b]	258 (27.3)	31 (33.3)	227 (26.6)	0.17	31 (33.3)	96 (34.4)	-0.0228
Solid organ transplant, n (%)	33 (3.5)	9 (9.7)	24 (2.8)	0.001	NA	NA	NA
Bone marrow transplant, n (%)	13 (1.4)	1 (1.1)	12 (1.4)	0.92	NA	NA	NA
ANC <500 cells/ul, n (%)	70 (7.4)	5 (5.4)	65 (7.6)	0.43	NA	NA	NA
ICU, n (%)	745 (78.8)	79 (85.0)	666 (78.1)	0.12	79 (85.0)	337 (85.0)	0.0000
Source controlled by day 14, n (%)[c]	752 (79.5)	69 (74.2)	683 (80.1)	0.18	69 (74.2)	213 (76.3)	-0.0491
Source of BSI, n (%)							
Intra-abdominal[d]	267 (28.2)	34 (36.6)	233 (27.3)	0.60	NA	NA	NA
Neutropenic fever[e]	18 (1.9)	2 (2.2)	16 (1.9)	0.85	NA	NA	NA

© 2024 Karaba SM et al. *JAMA Network Open*.

Prostatitis	2 (0.21)	1 (1.1)	1 (0.1)	0.06	NA	NA	NA
Respiratory	138 (14.6)	15 (16.1)	123 (14.4)	0.66	NA	NA	NA
Skin or soft tissue	57 (6.0)	5 (5.4)	52 (6.1)	0.78	NA	NA	NA
Urinary tract	337 (35.6)	25 (26.9)	312 (36.6)	0.06	25 (26.9)	73 (26.2)	0.0162
Vascular catheter	67 (7.1)	6 (6.5)	61 (7.2)	0.80	NA	NA	NA
Active empiric antibiotic therapy Day 1, n (%)	825 (87.2)	84 (90.3)	741 (86.9)	0.34	84 (90.3)	257 (92.11)	-0.0606
Microbiology of BSI							
E. coli	397 (42.0)	39 (41.9)	358 (42.0)	1.00	39 (41.9)	113 (10.5)	0.0291
K. pneumoniae	175 (18.5)	10 (10.8)	165 (19.3)	0.04	10 (10.8)	33 (11.8)	-0.0347
P. aeruginosa	102 (10.8)	12 (12.9)	90 (10.6)	0.49	12 (12.9)	33 (11.8)	0.0321

[a]Three patients were missing data for the sex variable.
[b]Severe immunocompromise was defined by at least one of the following: hematopoietic stem cell transplant in the prior 12 months or active treatment for graft-versus-host disease, solid organ transplant recipient, malignancy with active chemotherapy in the prior 3 months, neutropenia (absolute neutrophil count <500 cells/mm^3), HIV with CD4 count <200 cells/mm^3, or receipt of corticosteroids at a dose equivalent to 10 mg daily of prednisone for >14 days or other immunosuppressive therapy.
[c]Source control was needed and achieved (e.g., removal of infected catheter, drainage of fluid collection).
[d]Intra-abdominal includes intra-abdominal abscess, typhlitis, presumed translocation in patients with diarrhea or other intestinal issues, and hepato-biliary including hepatic abscess.
[e]Neutropenic fever with no other identified source
[f]NA = not applicable

© 2024 Karaba SM et al. *JAMA Network Open*.

Supplemental Table 2. Baseline characteristics of subgroup of patients with bacterial isolates with elevated beta-lactam MICs[a] and receipt of that beta-lactam, patients receiving extended-infusion versus intermittent infusion beta-lactam therapy for gram-negative bloodstream infections, before and after propensity-score matching

		**Full Cohort**			**Propensity-Score Matched Cohort**		
	Total n= 259	Extended infusion n=28	Intermittent Infusion n=231	P-value	Extended infusion n=28	Intermittent Infusion n=84	Standardized Mean Difference
Age, median (IQR)	70 (58-82)	62.5 (53-68.5)	71 (59-84)	0.003	NA[f]	NA	NA
Sex					NA	NA	NA
Male, n (%)	148 (57.1)	17 (60.7)	131 (56.7)	0.69	NA	NA	NA
Female, n (%)	111 (42.9)	11 (39.3)	100 (43.3)	0.69	NA	NA	NA
Pitt bacteremia score ≥4, n (%)	55 (21.2)	8 (28.6)	47 (20.4)	0.32	8 (28.6)	16 (19.1)	0.2108
Charlson comorbidity index ≥5, n (%)	28 (10.8)	6 (21.4)	22 (9.5)	0.06	6 (21.4)	12 (14.3)	0.1741
Severe immune compromise, n (%)[b]	80 (31.0)	14 (50.0)	66 (28.6)	0.02	14 (50.0)	35 (41.7)	0.1667
Solid organ transplant, n (%)	14 (5.4)	5 (17.9)	9 (3.90)	0.002	NA	NA	NA
Bone marrow transplant, n (%)	5 (1.9)	1 (3.6)	4 (1.7)	0.50	NA	NA	NA
ANC <500 cells/ul, n (%)	15 (5.8)	2 (7.1)	13 (5.6)	0.75	NA	NA	NA
ICU, n (%)	91 (35.3)	13 (46.4)	78 (33.9)	0.19	13 (46.4)	34 (40.5)	0.1194
Source controlled by day 14, n (%)[c]	219 (84.6)	20 (71.4)	199 (86.2)	0.04	20 (71.4)	59 (70.2)	0.0264
Source of BSI, n (%)							
Intra-abdominal[d]	82 (31.7)	13 (46.4)	69 (29.9)	0.08	NA	NA	NA
Neutropenic fever[e]	8 (3.1)	0 (0.0)	8 (3.46)	0.32	NA	NA	NA
Prostatitis	3 (1.2)	0 (0.0)	3 (1.3)	0.54	NA	NA	NA
Respiratory	17 (6.6)	0 (0.0)	17 (7.4)	0.14	NA	NA	NA
Skin or soft tissue	16 (6.2)	5 (17.9)	11 (4.8)	0.007	NA	NA	NA
Urinary tract	115 (44.4)	7 (25.0)	108 (46.8)	0.03	7 (25.0)	21 (25.0)	0.0000
Vascular catheter	8 (3.1)	1 (3.6)	7 (3.0)	0.88	NA	NA	NA

© 2024 Karaba SM et al. *JAMA Network Open*.

Active empiric antibiotic therapy Day 1, n (%)	192 (74.1)	17 (60.7)	175 (75.8)	0.09	17 (60.7)	52 (62.0)	-0.0244
Microbiology of BSI							
E. coli	126 (48.7)	7 (25.0)	119 (51.5)	0.008	7 (25.0)	20 (23.8)	0.0275
K. pneumoniae	57 (22.0)	5 (17.9)	52 (22.5)	0.58	5 (17.9)	18 (21.4)	-0.0933
P. aeruginosa	36 (13.9)	9 (32.1)	27 (11.7)	0.003	9 (32.1)	24 (28.6)	0.0765

[a]Defined as antibiotic MICs in the intermediate or susceptible dose-dependent range for the antibiotic administered.
[b]Severe immunocompromise was defined by at least one of the following: hematopoietic stem cell transplant in the prior 12 months or active treatment for graft-versus-host disease, solid organ transplant recipient, malignancy with active chemotherapy in the prior 3 months, neutropenia (absolute neutrophil count <500 cells/mm^3), HIV with CD4 count <200 cells/mm^3, or receipt of corticosteroids at a dose equivalent to 10 mg daily of prednisone for >14 days or other immunosuppressive therapy.
[c]Source control was needed and achieved (e.g., removal of infected catheter, drainage of fluid collection).
[d]Intra-abdominal includes intra-abdominal abscess, typhlitis, presumed translocation in patients with diarrhea or other intestinal issues, and hepato-biliary including hepatic abscess.
[e]Neutropenic fever with no other identified source
[f]NA = not applicable

© 2024 Karaba SM et al. *JAMA Network Open*.
