# Supplementary material for: Extended-Infusion β-Lactam Therapy, Mortality, and Subsequent Antibiotic Resistance Among Hospitalized Adults With Gram-Negative Bloodstream Infections
Source: JAMA Netw Open. 2024 Jul 2;7(7):e2418234. doi: 10.1001/jamanetworkopen.2024.18234 (PMC11220563; doi:10.1001/jamanetworkopen.2024.18234)
Supplement: Supplement 2. — Data Sharing Statement [file jamanetwopen-e2418234-s002.pdf]

# Data Sharing Statement

Karaba. Extended-Infusion β-Lactam Therapy, Mortality, and Subsequent Antibiotic Resistance Among Hospitalized Adults With Gram-Negative Bloodstream Infections. *JAMA Netw Open*. Published July 02, 2024. doi:10.1001/jamanetworkopen.2024.18234

**Data**
**Data available:** No
